# Historical Evolution of Unrelated Donor (URD) Recruitment of the Murcia Region Registry and Conditioning Social Events: Assessment of Quality, Effective URD Improvement, and Molecular HLA Characterization by NGS

**DOI:** 10.3390/ijms27188218

**Published:** 2026-09-15

**Authors:** Manuel Muro-Pérez, María Rosa Moya-Quiles, Carmen Botella, José Antonio Galián, Marina Fernández-González, Rosana González-López, María José Alegría, Alicia Hita, Helios Martínez-Banaclocha, Javier Muro, Isabel Legaz, Manuel Muro

**Affiliations:** 1 Immunology Service, University Clinical Hospital Virgen de la Arrixaca, Biomedical Research Institute of Murcia (IMIB), 30120 Murcia, Spain; muro_manuel@live.com (M.M.-P.); rosa.moya2@carm.es (M.R.M.-Q.); mcarmen.botella@carm.es (C.B.); josea.galian3@carm.es (J.A.G.); marina.fernandez3@carm.es (M.F.-G.); rosana.gonzalez@carm.es (R.G.-L.); mariaj.alegria@carm.es (M.J.A.); alicia.hita@carm.es (A.H.); helios.martinez2@carm.es (H.M.-B.); javimuro12@gmail.com (J.M.); 2 Department of Legal and Forensic Medicine, Biomedical Research Institute of Murcia (IMIB), Faculty of Medicine, Regional Campus of International Excellence “Campus Mare Nostrum”, University of Murcia, 30100 Murcia, Spain

**Keywords:** hematopoietic stem cell transplantation (HSCT), hematopoietic stem cell donor registry, HLA polymorphism, human leukocyte antigen (HLA), Immunogenetics, next-generation sequencing, novel HLA alleles

## Abstract

The Murcia Region unrelated donor (URD) registry was established in 1992 as part of the Spanish Bone Marrow Donor Registry (REDMO). This study aimed to comprehensively characterize the long-term evolution of URD recruitment, human leukocyte antigen (HLA) genetic diversity, allele and haplotype frequencies, and the spectrum of novel HLA alleles detected over the 34 years from 1992 to 2025. Recruitment trends, URD demographic characteristics, HLA allele and haplotype frequencies, and newly detected HLA alleles were analyzed retrospectively. HLA typing was performed using serological methods (1992–1996); PCR-SSO, PCR-SSP, and PCR-SSO Luminex (1997–2018); sequence-based typing (SBT, 2018–2021); and next-generation sequencing (NGS, 2021–2025). NGS was used to analyze a subset of 650 URDs to determine high-resolution HLA allele frequencies, Hardy–Weinberg equilibrium, expected heterozygosity, and extended HLA haplotypes. Newly detected HLA alleles were further characterized by the affected HLA locus and the localization of sequence variants within exons, introns, and the 3′-untranslated region (3′UTR). Between 1992 and 2025, 33.271 URDs were recruited, of whom 27.107 remained active in REDMO at the time of analysis. URD recruitment increased substantially following the implementation of the Spanish National Bone Marrow Plan in 2012 and reached a historical high in 2017. Additional recruitment peaks were associated with major regional public awareness campaigns. High-resolution HLA analysis of the NGS cohort demonstrated extensive immunogenetic diversity across all analyzed HLA loci, with a mean expected heterozygosity of 0.914. Ten frequent extended HLA haplotypes were identified, the most prevalent being HLA-A*29:02-C*16:01-B*44:03-DRB1*07:01-DQB1*02:02. Thirty-nine novel HLA alleles were characterized, exhibiting heterogeneous distributions among HLA loci and across coding and non-coding genomic regions. Exonic variants were predominantly detected in HLA-C, whereas intronic variants were mainly observed in HLA-C, HLA-DQB1, and HLA-A. This study integrates 34 years of URD recruitment with high-resolution immunogenetic characterization of the Murcia Region URD registry, providing new insights into the evolution of donor recruitment, HLA diversity, and the discovery of novel HLA alleles. The implementation of NGS enabled precise molecular characterization of HLA diversity and substantially expanded the detection and characterization of novel HLA alleles. Together, these findings provide a robust immunogenetic framework for the Murcia URD population, supporting donor recruitment strategies, optimization of URD searches, registry management, and future research in population genetics and hematopoietic stem cell transplantation.

## 1. Introduction

Allogeneic hematopoietic stem cell transplantation (HSCT) is a potentially curative treatment for many hematological malignancies and other severe hematological disorders. When an HLA-identical related donor is unavailable, transplantation from an unrelated donor (URD) is the preferred alternative. Successful HSCT depends on optimal compatibility between the donor and recipient. Therefore, high-resolution HLA typing at six classical loci (HLA-A, -B, -C, -DRB1, -DQB1, and -DPB1) is routinely performed to identify complete 12/12 matches and clinically relevant mismatches that may affect graft-versus-host disease (GVHD), graft failure, relapse, and overall transplant outcome [1,2,3,4,5].

Voluntary unrelated donor registries have played a fundamental role in providing compatible donors for patients requiring allogeneic HSCT for more than four decades [6,7]. The first international unrelated donor registry was established in London in 1974 by Shirley Nolan after her son Anthony was diagnosed with Wiskott–Aldrich syndrome. This initiative served as the model for the subsequent development of national and international donor registries worldwide, substantially increasing the availability of compatible donors for patients requiring HSCT [4,6,8,9,10,11].

In Spain, the official unrelated donor registry is the Spanish Bone Marrow Donor Registry (REDMO), established in 1991 by the Josep Carreras Foundation with authorization from the Spanish Ministry of Health and the National Transplant Organization (ONT). REDMO currently includes more than 600,000 voluntary donors recruited through regional donor registries and bone marrow banks throughout Spain [12,13]. Regional donor registries are integral to REDMO, supporting donor recruitment and providing valuable information on the immunogenetic characteristics of local populations. The voluntary URD registry of the Region of Murcia (southeastern Spain) was established in 1992 as part of REDMO and has accumulated 34 years of donor recruitment and immunogenetic data. During this period, HLA typing evolved from serological methods to PCR-based techniques, sequence-based typing (SBT), and, more recently, next-generation sequencing (NGS), which has markedly improved HLA typing accuracy and enabled the identification of previously undescribed HLA alleles [14,15].

Despite the growing importance of regional donor registries for donor selection and transplantation, comprehensive longitudinal studies that integrate donor recruitment, demographic characteristics, high-resolution HLA diversity, haplotype distribution, and characterization of novel HLA alleles remain scarce.

Therefore, this study aimed to provide a comprehensive 34-year characterization of the Murcia URD registry (1992–2025), covering donor recruitment trends, demographic characteristics, HLA allele and haplotype frequencies, high-resolution immunogenetic diversity, and the spectrum of novel HLA alleles identified after the implementation of NGS.

## 2. Results

### 2.1. Longitudinal Trends in URD Recruitment (1992–2025)

**Figure 1:**
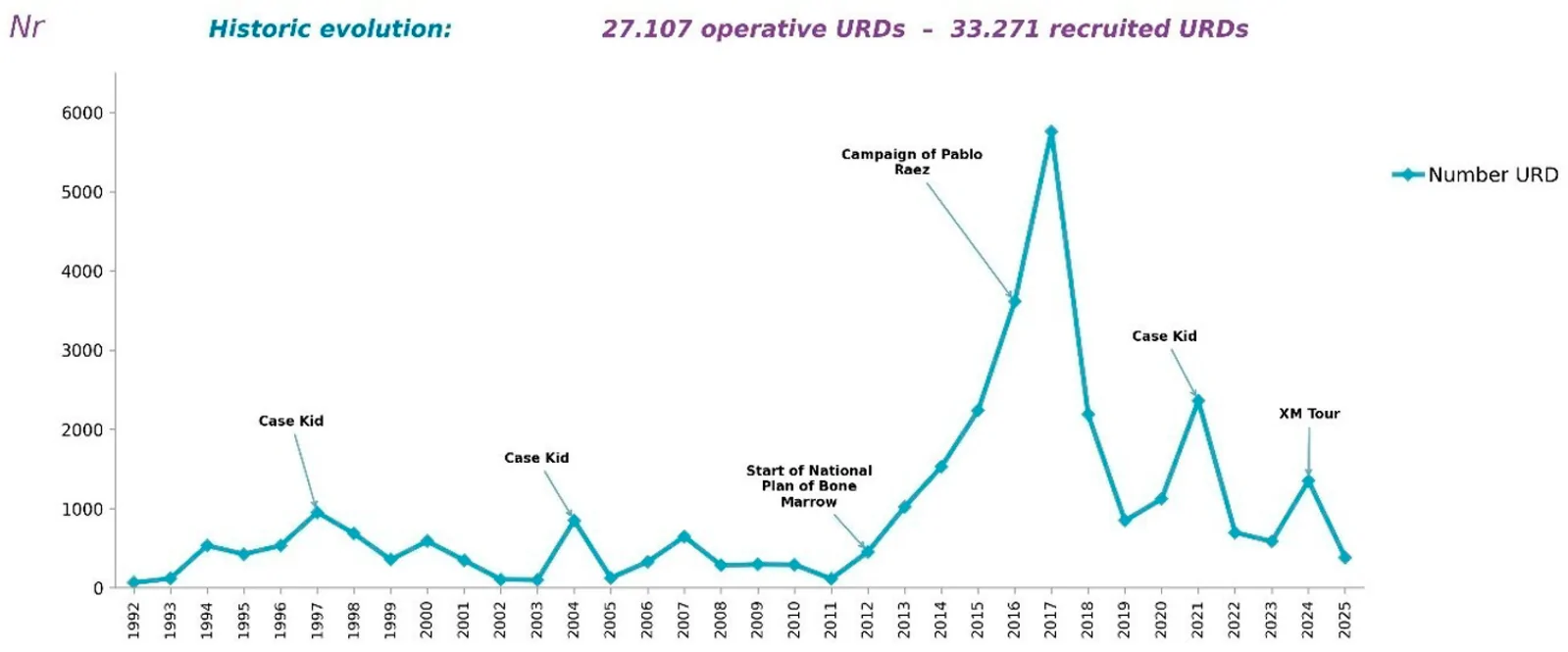
Historical evolution of unrelated donor (URD) recruitment in the Region of Murcia between 1992 and 2025. A total of 33,271 URDs were recruited during the study period, of whom 27,107 remained active in the Spanish Bone Marrow Donor Registry (REDMO) at the time of analysis after removal due to the age limit for donation and/or death. The graph illustrates annual recruitment trends and highlights the impact of major public awareness campaigns and institutional initiatives, including the Spanish National Bone Marrow Plan, on URD recruitment. Nr, number of recruited donors; REDMO, Spanish Bone Marrow Donor Registry; URD, unrelated donor; XM, crossmatch; XM Tour, crossmatch donor recruitment campaign.

The historical evolution of unrelated donor (URD) recruitment in the Region of Murcia between 1992 and 2025 is shown in Figure 1.

A total of 33.271 URDs were recruited. As in the 1st phase (1992–2012), URDs were accepted between ages 18 and 55. There has been a gradual decline in the number of URDs from earlier years due to reaching age limit and/or death, resulting in a difference of 6.164 URDs, leaving 27.107 active URDs in REDMO (Spanish URD Registry) at the time of this study. Regarding the distribution by year, we must highlight 1998 (685 URDs), 2004 (849 URDs), and 2008 (646 URDs), which saw increases due to public concern over a particular child’s leukemia/lymphoma case. Subsequently, there was a gradual decline until 2012, when, through ONT and REDMO, a new National Bone Marrow Plan was approved, increasing the number of recruited URDs (2013-1.020 URDs, 2014-1.527 URDs, 2015-2.237 URDs, and 2016-3.613 URDs). The highest number in history was reached in 2017, at 5.760 URDs, due to the recruitment campaign led by Pablo Raez, a young man with leukemia who battled the disease for two years and inspired the entire country to become URD. Then URD recruitment fell to 2.186 URDs in 2018. In 2020–2021, there was another increase (to 1.120 and 2.362 URDs) due to public social concerns with other particular child’s leukemia case, although the COVID-19 pandemic did not appear to affect the incorporation of new URDs. In 2024, due to the national Crossmatch Tour (XM-Tour) recruitment campaign, 1.352 URDs were recruited. This made the region the autonomous community that recruited the most URDs within the national campaign context. The Match Tour is an awareness campaign launched in September 2023 by the ONT, regional autonomous communities, and the “Fundación Josep Carreras contra la Leucemia” to promote bone marrow donation and boost registration in REDMO.

### 2.2. Demographic Characteristics and Geographical Distribution of URDs Recruited Between 1992 and 2025

Table 1 summarizes the demographic characteristics of unrelated donors (URDs) recruited between 1992 and 2025 by sex and age group (this data is collected right at the moment of recruitment). Overall, women represented a higher proportion of recruited URDs than men (63.4% vs. 36.6%). By age group, the 18–30-year group accounted for the largest share of donors (35.51%), followed by the 31–40-year group (32.06%), while donors aged 51–55 years account for the smallest share (11.30%).

**Figure 2:**
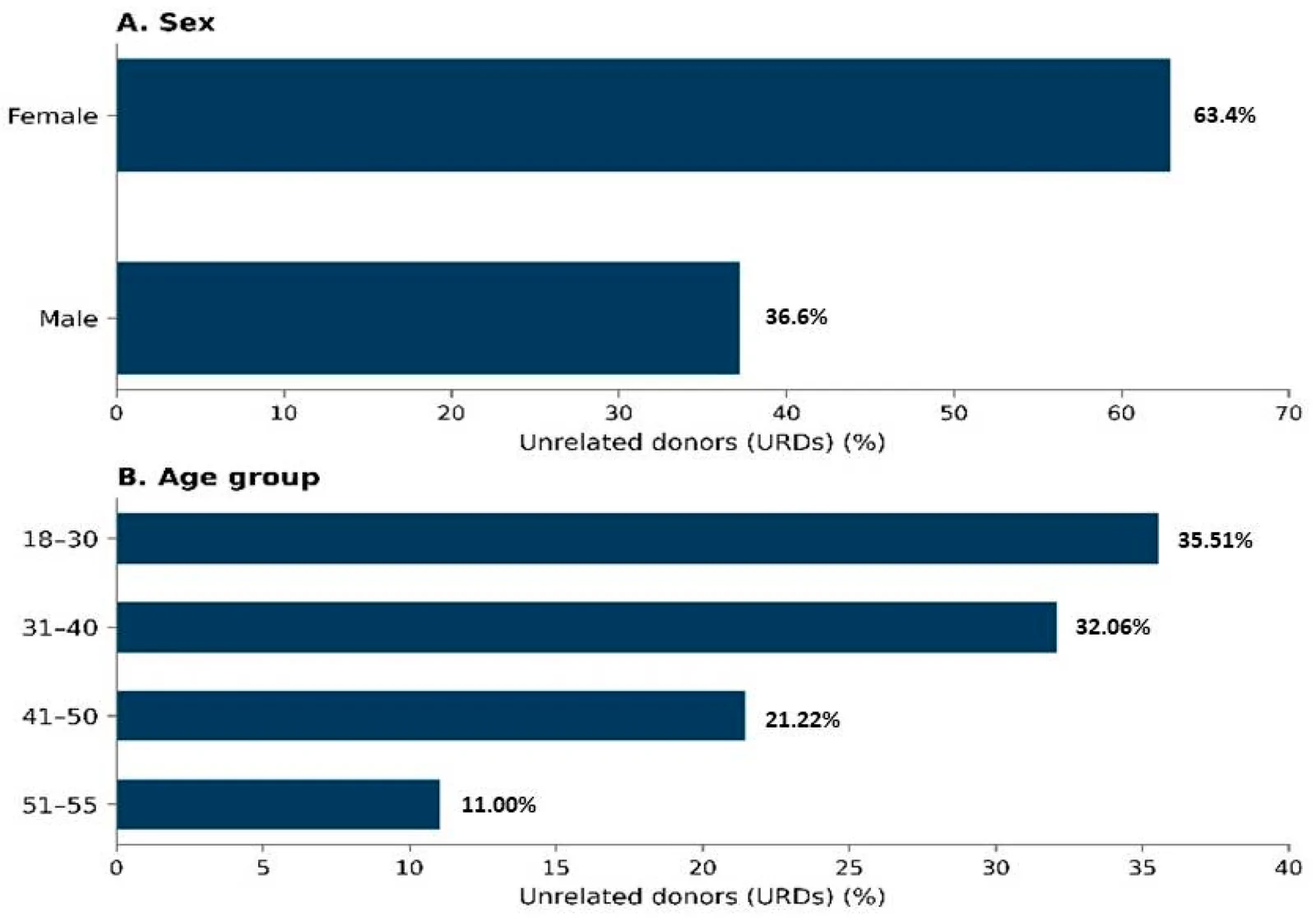
Distribution of unrelated donors (URDs) recruited between 1992 and 2025 according to sex (**A**) and age group (**B**). Panel A shows the sex distribution of recruited URDs, whereas Panel B shows the age distribution at the time of recruitment.

Figure 2 highlights the predominance of female donors and younger age groups among recruited URDs. The higher representation of donors aged 18–30 years is likely related to changes in donor recruitment policy in Spain. Since 2018, recruitment has focused primarily on younger individuals, whereas the inclusion of first-time donors aged 41–55 years has no longer been permitted. Consequently, younger age groups have accumulated a longer recruitment period, which may partially explain their greater representation in the registry.

**Figure 3:**
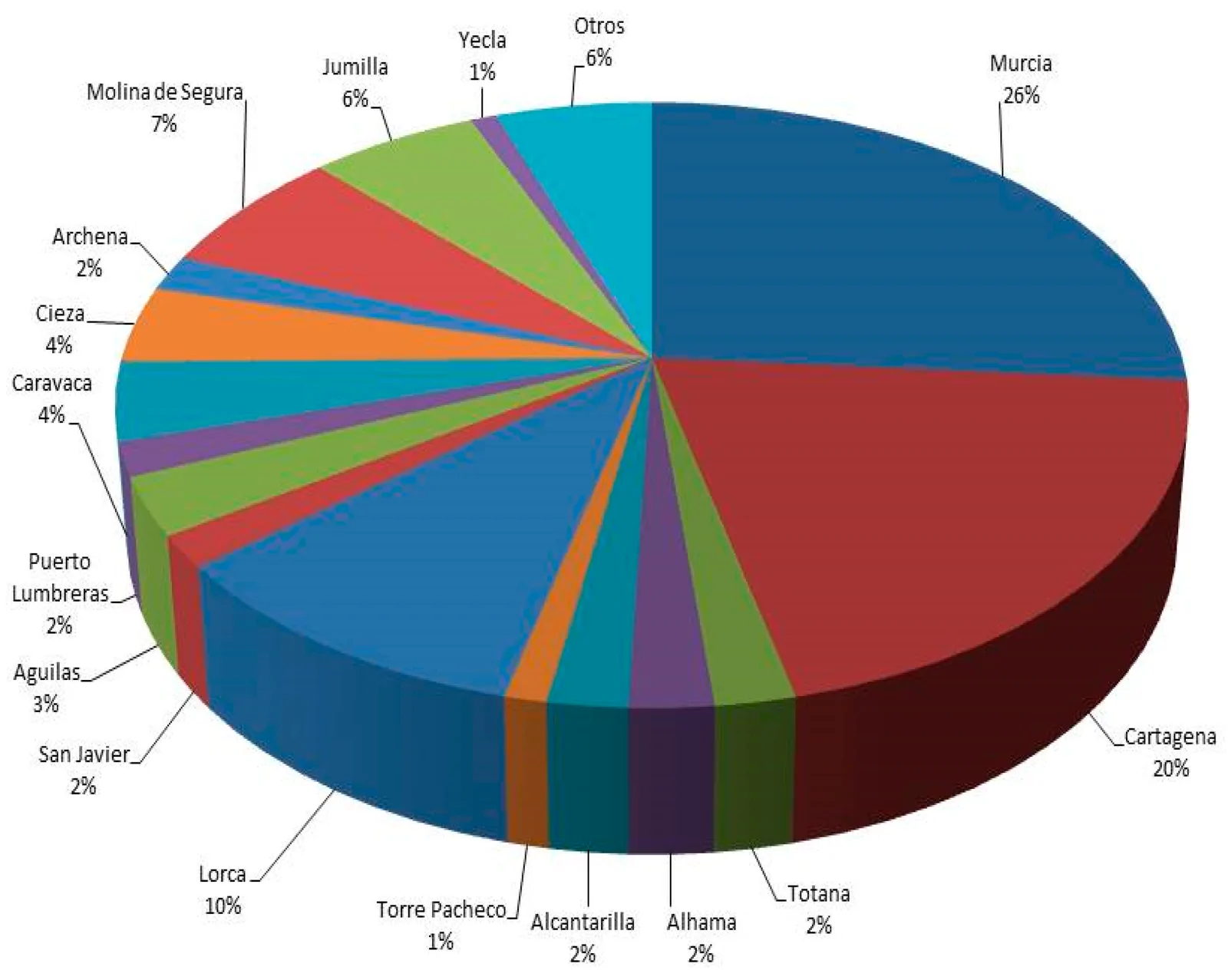
Geographical distribution of unrelated donors (URDs) recruited in the Region of Murcia between 1992 and 2025.

The geographical distribution of recruited URDs across the municipalities of the Region of Murcia is shown in Figure 3. The figure cited refers to the municipality, which may encompass both the city and rural areas. In fact, the informed consent form signed by our URDs does not specify any requirements regarding residence in rural or urban areas, economic specialization, social status, or educational attainment.

The largest proportions of donors were recruited in the metropolitan municipalities of Murcia (26%) and Cartagena (20%), which together accounted for approximately 46% of all registered URDs. Lorca contributed an additional 10% of recruited donors, followed by Jumilla (7%).

**Figure 4:**
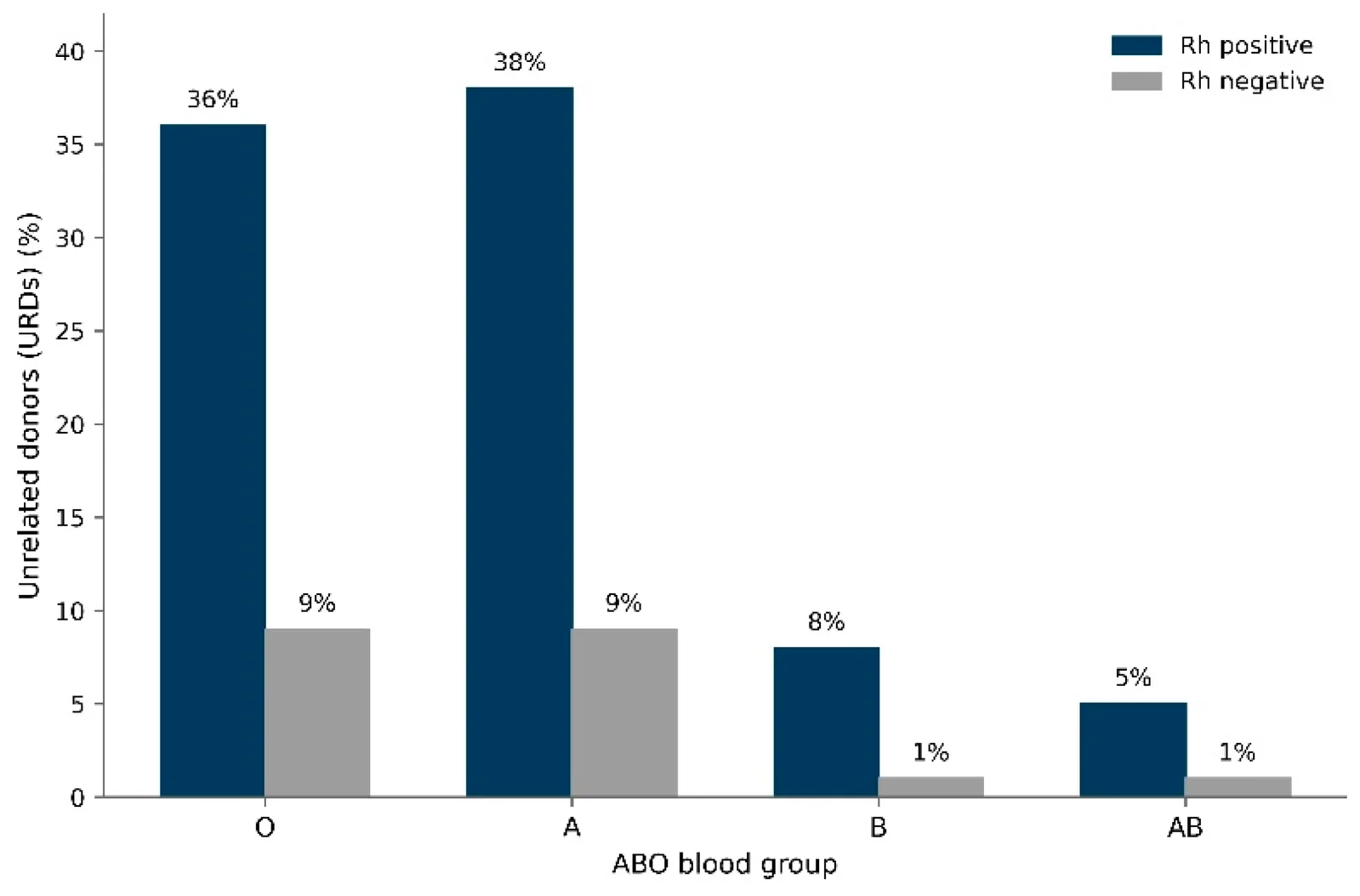
Distribution of ABO and Rh blood groups among unrelated donors (URDs) recruited in the Region of Murcia.

The distribution of ABO and Rh blood groups among our recruited URDs is presented in Figure 4. Blood groups O-positive and A-positive were the most frequent, consistent with the ABO and Rh distribution reported for the general Spanish population.

### 2.3. Evolution of National and International Requests for HLA Typing Extension, Confirmatory Samples, and Stem Cell Collections for Transplant (2012–2025)

**Figure 5:**
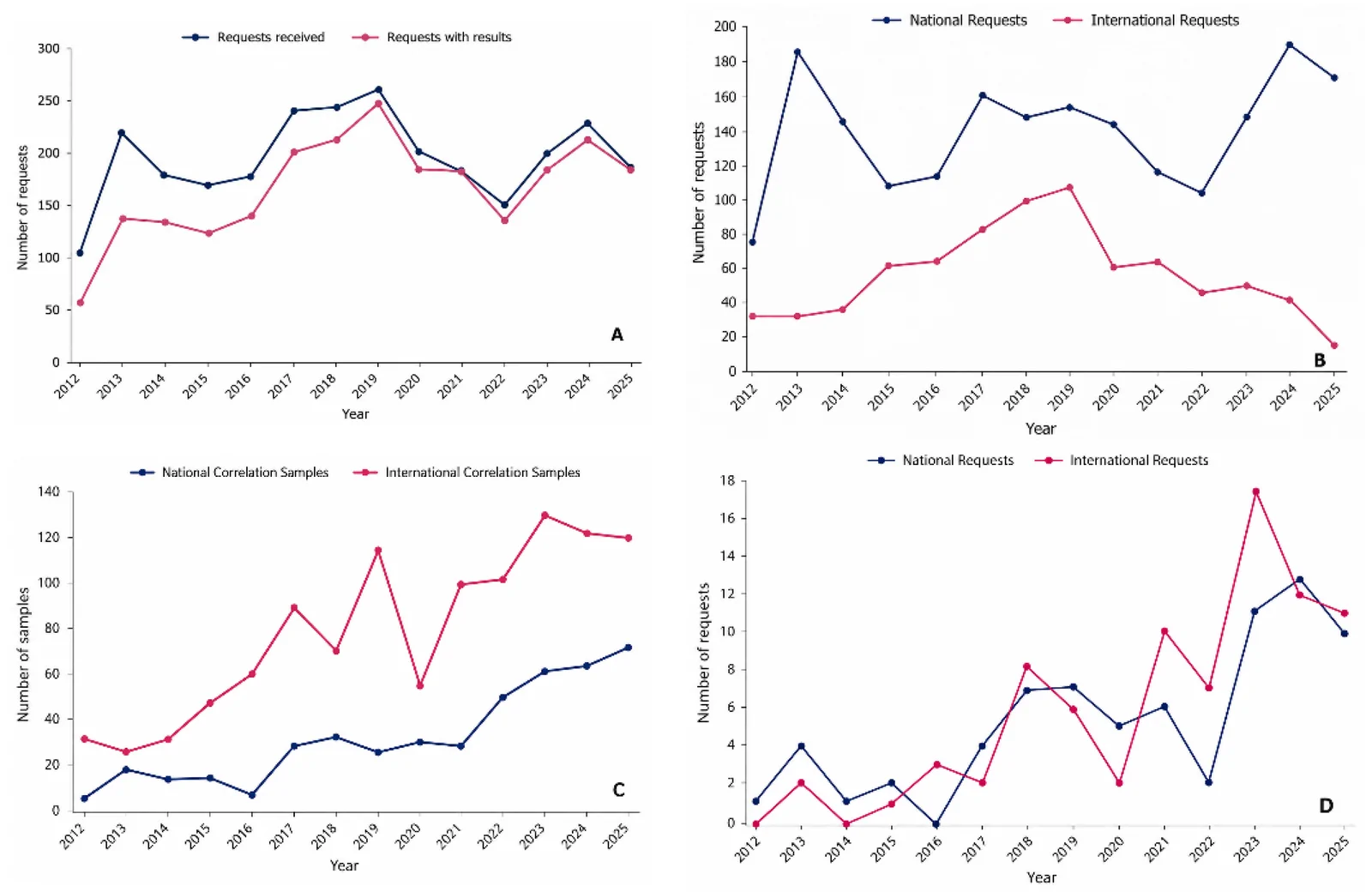
Temporal evolution of national and international requests involving unrelated donors (URDs) registered in the Murcia Registry between 2012 and 2025. (**A**) Donor search requests. (**B**) Requests for HLA typing extension or recheck. (**C**) Confirmatory sample requests. (**D**) Final hematopoietic stem cell collections were performed for transplantation.

The evolution of national and international requests involving unrelated donors (URDs) from the Murcia Registry between 2012 and 2025 is summarized in Figure 5. Overall, international registries generated more requests than national registries throughout the study period. This trend was consistently observed across donor searches, HLA typing extension or recheck requests, confirmatory sample requests, and final hematopoietic stem cell collections for transplantation.

Figure 5A presents the annual number of combined national and international requests for URDs received by the Murcia Registry. Figure 5B shows the distribution of national and international requests for HLA typing extension or recheck received from other registries. Figure 5C illustrates the annual number of national and international requests for confirmatory (verification) samples from URDs registered in the Murcia Registry. Finally, Figure 5D presents the number of final hematopoietic stem cell collections performed for hematopoietic stem cell transplantation (HSCT), distinguishing between national and international collections. Overall, international registries generated more requests than national registries across all analyzed activities, except in the case of typing extension or recheck received where the situation was the reverse (Figure 5B). This makes sense, given that expanded HLA typing for Spanish donors is provided free of charge among the country’s regional registries.

### 2.4. Genetic Structure of the HLA System in the Murcia Unrelated Donor Population

A total of 650 unrelated donors [URDs typed exclusively by next-generation sequencing (NGS)], were included in this analysis. The rest of URDs (which were typed using methods other than NGS) were not considered in this part of our study. HLA allele and haplotype frequencies were assessed at full resolution for the HLA-A, -B, -C, -DRB1, -DQB1 and -DPB1 loci.

Our population’s genetic structure was assessed by comparing observed and expected genotype frequencies under Hardy–Weinberg equilibrium (HWE). No statistically significant deviations from HWE were observed at the HLA-A, -B, -C, -DRB1, and -DQB1 loci (all *p* > 0.05). In contrast, the HLA-DPB1 locus showed a slight but statistically significant deviation from HWE (*p* = 0.035) (Table 2).

Expected heterozygosity values for each HLA locus are summarized in Table 3. Heterozygosity ranged from 0.8532 at the HLA-DPB1 locus to 0.9643 at the HLA-B locus, reflecting the high genetic diversity across the HLA system. The overall mean expected heterozygosity across all loci was 0.9138.

Allele frequencies at HLA class I and class II loci are presented in Table 4 and Table 5, respectively. Among HLA class I loci (Table 4), 33 distinct alleles were identified at HLA-A, 45 at HLA-B, and 26 at HLA-C loci, considering only alleles detected in at least two URDs or in one URD with a clear four-digits difference. For HLA class II (Table 5), 31 distinct alleles were identified at HLA-DRB1, 20 at HLA-DQB1, and 31 at HLA-DPB1 loci. Among HLA class I loci, HLA-A*02:01 was the most frequent allele (30.46%), whereas HLA-B*44:02 (10.1%) and both HLA-C*04:01 and HLA-C*07:01 (14.1% each) were the predominant alleles at HLA-B and HLA-C loci, respectively.

Among HLA class II loci, HLA-DPB1 showed a marked predominance of the HLA-DPB1*04:01P allele, which accounted for 35.3% of all HLA-DPB1 alleles (Table 5). The high frequency of this allele may have contributed to the reduced heterozygosity and the slight deviation from Hardy–Weinberg equilibrium observed at this locus. Overall, the allele frequency distributions in the Murcia unrelated donor population were consistent with those previously reported for other Spanish populations.

Among the 2.850 possible extended HLA-A/B/C/DRB1/DQB1 haplotypes identified in the study of haplotype frequencies only 10 were observed in our population, with each one present in at least 2 URDs who carried it, and also supported by a previous study of ours several years ago [16]. These were considered the most frequent HLA haplotypes detected in our population of URDs from the Murcia Region. The distribution of these 10 haplotypes is shown in Table 6.

The 4 most frequent haplotypes account for 71.5% of the total across all 10 haplotypes found in our population, suggesting that haplotypes of Celtic, Ibero-Paleo-North African, and Mediterranean origin make up more than 70%, reflecting the likely historical origin of the inhabitants of our region, as will be argued in Section 3.

### 2.5. New Alleles Described in the Population of URDs of Murcia Region Between 1992 and 2025 and Localization in Exon, Intron or 3’UTR

During the study period (1992–2025), 39 officially designated novel HLA alleles were identified in unrelated donors from the Murcia Registry. These alleles included sequence variants affecting both coding (exonic) and non-coding (intronic and 3′UTR) regions [14,15,16,17]. The WHO Nomenclature Committee for Factors of the HLA System subsequently assigned official nomenclature for these discovered alleles.

**Figure 6:**
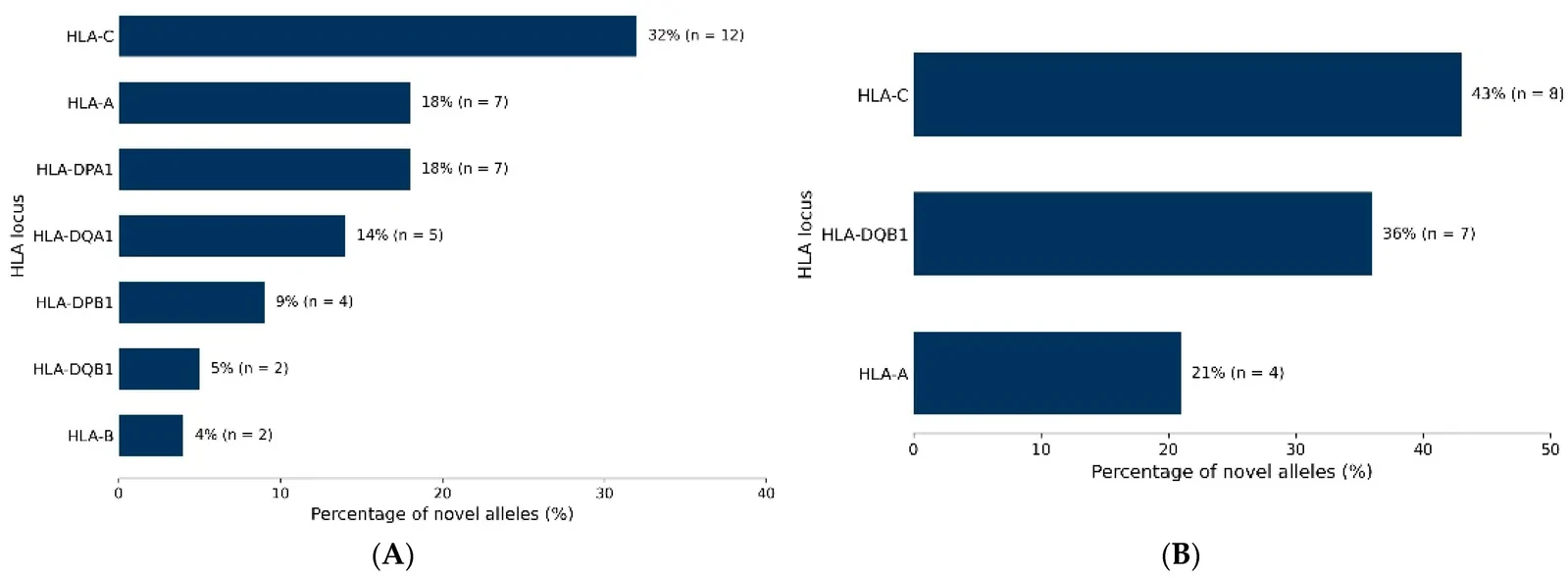
Distribution of newly identified HLA alleles according to HLA locus in the Murcia Region unrelated donor (URD) population between 1992 and 2025. (**A**) Novel HLA alleles carrying exonic sequence variants. (**B**) Novel HLA alleles carrying intronic sequence variants.

First, Figure 6 shows the distribution of previously reported HLA alleles detected in the Murcia Registry, stratified by HLA locus and by the genomic location of the sequence variants. Among alleles with exonic variants (Figure 6A), HLA-C had the highest proportion (32%), followed by HLA-A and HLA-DPA1 (18% each), HLA-DQA1 (14%), HLA-DPB1 (9%), and HLA-B and HLA-DQB1 (5% each). No exonic variants were detected at the HLA-DRB1 locus. Among alleles with intronic variants (Figure 6B), HLA-C locus again had the highest proportion (43%), followed by HLA-DQB1 (36%) and HLA-A (21%) loci. No intronic variants were observed at the remaining loci.

**Figure 7:**
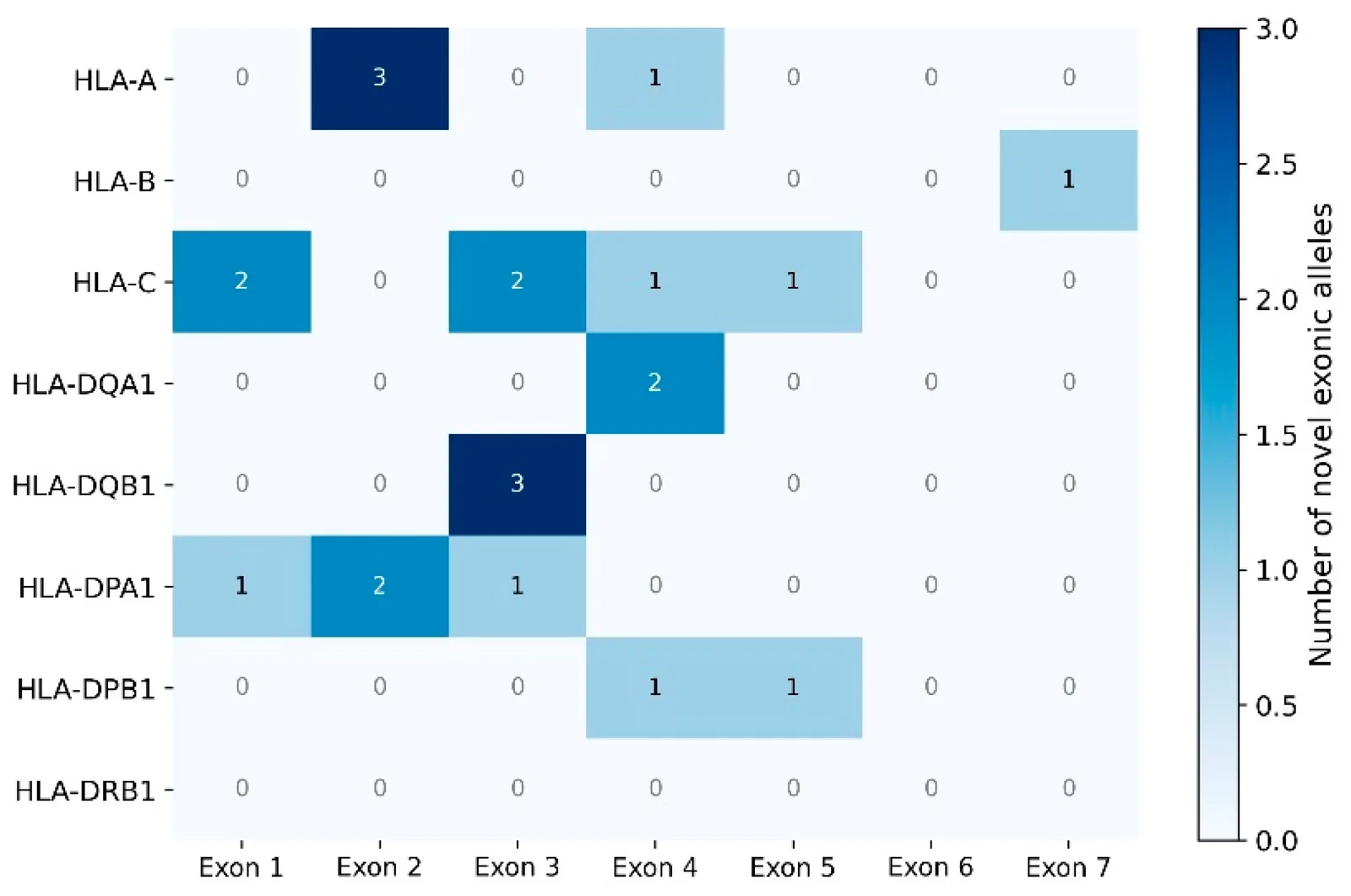
Distribution of newly identified HLA alleles carrying exonic sequence variants across HLA loci according to the affected exon in the Murcia Region unrelated donor (URD) population (1992–2025). Cell values indicate the number of novel alleles identified for each HLA locus and exon.

Secondly, Figure 7 summarizes the distribution of exonic sequence variants among previously reported HLA alleles detected in the Murcia Registry. Variants were identified across multiple exons depending on the HLA locus. HLA-A variants were primarily located in exon 2, whereas HLA-C variants were distributed across exons 1, 3, 4, and 5. Variants affecting HLA-DQA1 were located exclusively in exon 4, whereas all HLA-DQB1 variants were in exon 3. HLA-DPA1 variants were identified in exons 1–3, whereas HLA-DPB1 variants affected exons 4 and 5.

**Figure 8:**
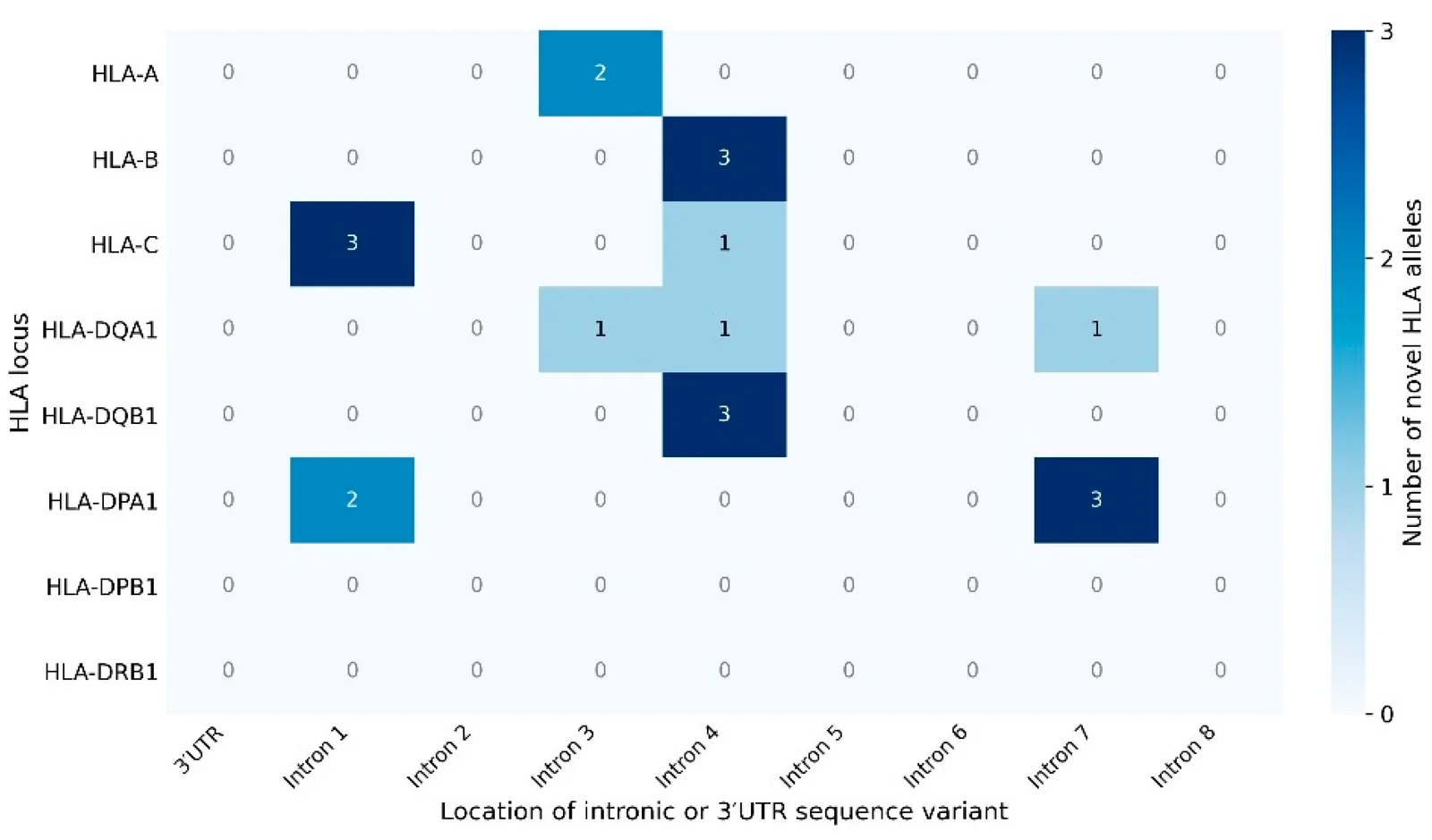
Distribution of newly identified HLA alleles according to the location of intronic and 3′UTR sequence variants in the Murcia Region unrelated donor population (1992–2025). Cell values indicate the number of novel alleles identified at each HLA locus and genomic region.

Thirdly, Figure 8 shows the distribution of intronic and 3′UTR sequence variants among previously reported HLA alleles detected in the Murcia Registry. Intronic variants were identified at HLA-A, HLA-C, and HLA-DQB1, whereas 3′-UTR variants were observed at the corresponding loci indicated in the figure.

## 3. Discussion

This study presents the first comprehensive longitudinal characterization of the Region of Murcia’s unrelated donor (URD) registry over 34 years (1992–2025). Beyond describing donor recruitment trends, it integrates demographic characteristics, high-resolution HLA diversity, allele and haplotype frequencies, and the detection of previously reported HLA alleles in this population following the implementation of next-generation sequencing (NGS). Together, these findings provide an updated overview of the immunogenetic characteristics of the Murcia donor registry and their potential relevance to donor selection and hematopoietic stem cell transplantation.

The temporal evolution of donor recruitment shows that registry growth depends not only on technical advances in HLA typing but also on coordinated national recruitment strategies and public engagement. The marked increase observed after the implementation of the Spanish National Bone Marrow Plan supports the effectiveness of organized national initiatives to expand the unrelated donor pool.

The sustained increase in donor recruitment since 2012 aligns with the main objectives of the Spanish National Bone Marrow Plan, which seeks to expand the REDMO registry [12], through coordinated recruitment campaigns and improved national organization. Similar increases have been reported in other national donor registries after the implementation of structured recruitment policies, underscoring the importance of sustained institutional support to maintain an adequate donor pool.

Similarly, adapting NGS technology to meet REDMO’s entry requirements, the need to implement it, and lowering the age limit for inclusion to under 40 (with a possible further reduction to under 35 in the near future) have increased the number of successful collections in our registry.

Furthermore, immunogenetic characterization, such as estimating our HLA allelic and haplotypic frequencies, can expedite searches for unrelated donors for patients with uncommon profiles. Additionally, from a new socio-health perspective, it can facilitate understanding of the events that led to significant fluctuations in our registry’s activity and enable the development of new measures to improve the management of our unrelated donor bank (Figure 1).

From a socio-health perspective, this study identifies three key events that significantly affected the annual number of donors recruited: the 2012 National Bone Marrow Donation Plan [12], Pablo Ráez’s (a patient with leukemia) 2016 social media campaign [18], and the 2018 policy prioritizing the selection of new donors under age 40 [8]. The observed increases and decreases in donor numbers are consistent with REDMO’s published comparisons with other national bone marrow banks and registries [19].

On the other hand, campaigns organized by the ONT, REDMO, and our regional transplant coordination office, such as the “Crossmatch Tour,” have also expanded our registry, as shown in Figure 1. Notably, our autonomous community has historically ranked second in unrelated donors per 1.000 inhabitants, surpassed only by Navarre [19,20].

Since January 2018, in clinical practice, the criteria for selecting an optimal bone marrow donor for onco-hematological patients have required a 10/10 match at the high-resolution level across the five main HLA loci (HLA-A, -B, -C, -DRB1, and -DQB1), because any major or minor mismatch is associated with poorer HSCT outcomes [21].

In this regard, transplant centers now routinely include HLA-DPB1 locus typing, given studies indicating that mismatching at this locus may increase the risk of acute graft-versus-host disease (aGVHD) [11,22,23,24,25]. In this context, the most notable findings of our study concern HLA-DPB1; the most frequent alleles observed in our URD population were DPB1*04:01P, DPB1*04:02, DPB1*02:01P, and DPB1*03:01 (see Table 5). These results align with those reported by Shaw et al. [26] for a Caucasian population and are similar to those reported by Morishima et al. [23] for Japanese population.

However, for extended haplotypes that include HLA-DPB1 in addition to the five classical loci, the genetic distance between HLA-DPB1 and HLA-DQB1 is greater than that between other HLA system genes that exhibit linkage disequilibrium due to their proximity. Furthermore, a recombination hotspot exists between HLA-DPB1 and HLA-DQB1, making it difficult to find haplotypes spanning these six loci at high frequency in any study population and even harder to find donors with 12/12 HLA identity with the patient [27]. Consequently, we did not include this locus in our study of the most frequent haplotypes in our population.

Next, we discuss the ancestral origins of the most common haplotypes observed in our region’s registry of unrelated donors.

The most frequent haplotype in our registry is A*29:02-C*16:01-B*44:03-DRB1*07:01-DQB1*02:02. Although it is among the most common across the Iberian Peninsula [28,29,30,31], it is also found among Danes, French, Italians, Germans, and Irish [32]. It has been described as a Western European haplotype, although it is also present among Americans of European ancestry, including Mexicans [27].

The second most frequent haplotype in our registry, A*01:01-C*07:01-B*08:01-DRB1*03:01-DQB1*02:01, is found throughout Northern, Central, and Eastern Europe. Haplotype frequencies similar to ours are observed in Portuguese, British, Danish, Swiss, Italian, Czech, Hungarian, Finnish, Greek, and Cretan populations. By contrast, higher frequencies are observed among Germans, Austrians, Croats, and Serbs [28,32,33,34]. This distribution has led to its classification as an ancient pan-European haplotype of Indo-European-Celtic origin.

The third most common haplotype in the Murcia URD registry was A*30:02-C*05:01-B*18:01-DRB1*03:01-DQB1*02:01. This haplotype is infrequent in European populations, except among Sardinians, where it occurs at high frequency [32,34] and is also common among Basques and Spaniards [29,35]. Interestingly, this haplotype is the most frequent in the Algerian population [33,36], leading to its characterization as Ibero-Paleo-North African in origin. However, within the Iberian Peninsula, this haplotype does not occur at significant frequencies among the Portuguese population; this suggests that this population may have remained genetically isolated from the Spanish, possibly due to limited historical interaction between the Portuguese and the Spanish (except in Galicia region), despite their being neighbors [33,37].

The fourth most common haplotype in our unrelated donor registry was A*33:01-C*08:02-B*14:02-DRB1*01:02-DQB1*05:01. This haplotype has the highest frequency in the Armenian population, leading to suggestions of a hypothetical Armenian origin [28]. It also shows very high frequencies across Mediterranean countries in Europe and Africa (Sardinians, French, Greeks, Algerians, and Italians) [28,32,34], while its frequency decreases as one moves toward Northern Europe. The frequency in our registry does not differ from that observed in Spanish or Algerian populations [33,34]. However, it appears to be absent among Basque, Catalan, and Portuguese populations, reflecting a separation from the Mediterranean area [29,38]. This is difficult to explain in the case of Catalans, given their classic Mediterranean profile. However, a recent study published by the Catalan Bone Marrow Donor Registry reports a haplotype frequency of up to 2.86 [39], suggesting that the earlier studies by Comas et al. [38] may have been somewhat biased.

The fifth most common haplotype in our unrelated donor registry, A*03:01-C*07:02-B*07:02-DRB1*15:01-DQB1*06:02, appears to be of pan-European origin, although its consistent presence across populations has not been widely documented [29].

The sixth most frequent haplotype among URDs in the Murcia region was A*02:01-C*07:02-B*07:02-DRB1*15:01-DQB1*06:02; this haplotype shows similar or higher frequencies in other Spanish populations, such as the Basques, as well as in Portuguese, French, and Breton populations [29,33,36]. Although it is also found among Germans, Finns, the Cornish population, Austrians, Algerians, Cretans, and other Mediterranean populations [33,36], it does not appear at high frequencies in Northern Europeans, such as Scandinavians. In this regard, it has been hypothesized that its origin is of the European–Basque–North African type [28,35].

The seventh most frequent haplotype in the URD population of the Murcia region was A*02:01-C*16:01-B*44:03-DRB1*07:01-DQB1*02:02. As the fifth haplotype, its origin has not been widely documented. This haplotype has been reported in Austrians, Italians, and Swiss [40,41], which could suggest a pan-European origin, or given its presence in other populations in the Spanish Levant, such as Catalans and Valencians, a possible Levantine-Catalan Mediterranean origin [38,42].

The eighth haplotype identified in our URD population was A*26:01-C*12:03-B*38:01-DRB1*13:01-DQB1*06:03. This haplotype appears at similar frequencies among the Portuguese [36,42] but is absent in other populations of the Iberian Peninsula. This pattern has led to the suggestion of shared ancestry between the Iberian populations of Murcia and Portugal, possibly linked to the ancient Kingdom of Tartessos (which extended from Olisipo, ancient Lisbon, to Mastia, ancient Cartagena). However, a possible Jewish origin might also explain this curious geographical distribution, as this haplotype is also found among Ashkenazi Jews (from Central and Eastern Europe) [34]. In this regard, there is considerable documentation of Jewish settlement in our autonomous community during the Middle Ages, specifically around the old “Puerta de Orihuela” Jewish quarter in the center of the region’s capital, as previously reported [16,29].

The ninth most frequent haplotype in our URD registry was A*02:01-C*03:03-B*15:01-DRB1*04:01-DQB1*03:02. This haplotype is found in Spaniards (Catalans and Basques) [35,42] and is also present in the Cornish population, among Finns, and in Australians and white Americans [29,40]. Curiously, this haplotype is very common among Inuit (Eskimo) populations and is shared with East Asian populations, including Japanese, Korean, and Chinese populations from central parts of the country [26,43,44].

Finally, the tenth most frequent haplotype in URDs from the Region of Murcia, albeit at a somewhat lower frequency, was A*02:01-C*05:01-B*18:01-DRB1*03:01-DQB1*02:01; we found no publications regarding its possible origin.

This study has limitations inherent to its retrospective design, as variables changed over the period from 1992 to 2025, spanning approximately 33 years. A primary limitation is the evolution of HLA typing methods: from basic serological CDC techniques to molecular methods such as PCR-SSP, PCR-SSO, SBT, and, more recently, NGS [45,46]. This shift resulted in final typing data, whether serological or molecular, being reported using varying nomenclatures (serological vs. low-resolution molecular vs. high- or ultra-resolution molecular), which occasionally necessitated the exclusion of samples that could not be compared across analyses [47]. For instance, serological B15 variants (B62, B63, B70, B71, or B72) can be difficult to map to their corresponding high-resolution molecular equivalents (e.g., B*15:01, B*15:10, B*15:17). Consequently, the frequency analysis was restricted to the 650 individuals for whom the main loci were typed at high resolution (at least 4 molecular digits).

Another limitation is the number of HLA loci typed over time: initially, in 1992, only HLA class I loci (HLA-A and -B) were typed; this was subsequently expanded to primarily three loci (HLA-A, -B, and -DR), then to five loci (HLA-A, -B, -C, -DRB1, and -DQB1), and most recently to eleven loci (HLA-A, -B, -C, -DRB1, -DRB3, -DRB4, -DRB5, -DQA1, -DQB1, -DPA1, and -DPB1). This progression made it difficult to obtain a more representative and complete sample size, which would have allowed us to increase the final number of URDs included in the analysis for this study and to characterize the regional registry for all loci involved.

Furthermore, minor limitations arose in the categorization of certain variables in the serial analysis of URD samples, including their origin, the transfer of donors between national registries, variations in discharge date formats, and missing key parameters, necessitating modifications, extensive analysis, or even the recording of variables.

In this context, and in light of the new challenges and objectives set by ONT, and the REDMO National Bone Marrow Plan, such as the potential lowering of the age limit (to 35 years), the possible use of buccal swabs for donor recruitment, and the prospect of having HLA typing (via NGS), blood group, and CMV status data available at the initial recruitment stage, an increase in the volume and quality of donor typing is anticipated. This includes retyping existing donors in our registry to facilitate the search for suitable, effective donors and to confirm compatibility with oncohematology patients more rapidly than is currently possible.

From a socio-health perspective, the objectives set by REDMO aim at closer and more efficient management of the various existing regional registries, such as the Murcia registry, to promote higher quality hematopoietic stem cell transplantation for each patient, while applying certain principles of the biopsychosocial approach and the post-personalized medicine movement [48,49].

In conclusion, we have a regional BMD bank whose URD count per 1.000 inhabitants ranked second highest in our country during 2015–2017 and, at the beginning of 2026, ranked among the top three regions in Spain. Today it ranks first. All HLA loci are in HWE and show heterozygosity, with a mean of 0.91, confirming the high genetic diversity of our registry. In this sense, thirty-three alleles were identified in HLA-A, 45 in HLA-B, 26 in HLA-C, 31 in HLA-DRB1, 20 in HLA-DQB1, and 31 in HLA-DPB1, each appearing in at least two individuals. The most frequent haplotype in our URDs was A*29:02-C*16:01-B*44:03-DRB1*07:01-DQB1*02:02, and the 4 main haplotypes accounted for 68.8% of the 10 most frequent, suggesting a pan-European, Celtic, Mediterranean & Ibero-Paleo-North African footprint in our regional genetic composition. In addition, 39 new HLA alleles were defined, mostly by NGS (*n* = 38). The locus with the newest exonic variants was HLA-C (32%, exons 1 and 3), followed by HLA-A (exons 2 and 4) and HLA-DPA1 (18%), HLA-DQA1 (14%), HLA-DPB1 (9%), and HLA-B and HLA-DQB1 (4%). Intronic variants were observed in HLA-C (43%), -DQB1 (36%) and -A (21%), with a particular presence of the 3’UTR region and intron 5 in HLA-C, intron 2 in HLA-DQB1, and intron 3 in HLA-A.

This study has additional important limitations that should be noted. Further research, including a large, well-characterized cohort and more thorough follow-up, will be required to validate our results in future analyses. Finally, increasing the number of donors is strictly necessary to corroborate these preliminary results. To observe the ultimate impact of the NGS technique on quality improvements, longer follow-up periods are also necessary.

## 4. Methods

### 4.1. Study Design, Technical Parameters, and Demographic Information

This retrospective observational study included voluntary unrelated hematopoietic stem cell donors recruited through the Murcia Regional Registry between January 1992 and December 2025 as part of the Spanish Bone Marrow Donor Registry (REDMO). Retrospective review covered demographic characteristics, annual recruitment data, HLA typing results, requests from national and international registries, confirmatory samples, hematopoietic stem cell collections, and novel HLA alleles identified during the study period.

HLA typing methods evolved during the study period in response to technological advances and REDMO requirements. Serological typing using complement-dependent cytotoxicity (CDC) was performed from 1992 to 1996. Molecular typing using polymerase chain reaction with sequence-specific oligonucleotides (PCR-SSO), sequence-specific primers (PCR-SSP), and PCR-SSO combined with Luminex technology was used from 1997 to 2018. Peripheral blood genomic DNA was isolated using a QIAsymphony SP system (Qiagen GmbH, Hilden, Germany) ac-cording to the manufacturer’s instructions. Sequence-based typing (SBT) was implemented from 2018 to 2021 [14], followed by next-generation sequencing (NGS) from 2021 to 2025, as previously described [15,50,51], using commercially available NGSgo Kits (GenDx, Utrecht, The Netherlands) on a MiSeq platform (Illumina, San Diego, CA, USA). Data were analyzed with NGS engine software (GenDx) version 4.1 and the 3.58.0 version of the IPD-IMGT/HLA Database.

For population genetic analyses, a subset of 650 unrelated donors typed exclusively by NGS was selected to ensure homogeneous, high-resolution HLA genotyping and to minimize potential bias associated with the different typing methods used throughout the study period.

All patients provided informed consent for inclusion in the study, in accordance with the protocols of ONT and REDMO. The present study was conducted in accordance with the Declaration of Helsinki, and the protocol was approved by the Ethics Committee of University Hospital HCUVA (PI15/01370).

### 4.2. Genetic Structure of Murcians URDs and Statistical Analysis

Population genetic analyses were conducted using Arlequin version 3.5.2.2 [52]. Allele frequencies observed and expected heterozygosity, Hardy–Weinberg equilibrium, and extended HLA-A~C~B~DRB1~DQB1 haplotype frequencies were estimated in a cohort of 650 unrelated donors typed exclusively by NGS technique. The rest of the URDs (which were typed using methods other than NGS) were not considered in this part of our study.

Deviations from Hardy–Weinberg equilibrium were assessed for each HLA locus using the exact test implemented in Arlequin [53].

When data originate from diploid individuals and the gametic phase is unknown (i.e., it is not known which allele is paired with which on the same chromosome), Arlequin uses advanced statistical algorithms to infer the most probable haplotypes and their frequencies. The program primarily implements two automated methods within its intra-population options [52]. The expectation–maximization (EM) algorithm was used in this study. This maximum-likelihood method estimates haplotype frequencies by iteratively resolving the ambiguity of heterozygous genotypes until the optimal estimate is reached [54]. In this manner, possible extended haplotype frequencies were estimated using this EM algorithm implemented in the same software. Haplotypes detected in at least two unrelated donors were included in the corresponding frequency tables.

Statistical analyses and graphical representations were conducted using IBM SPSS Statistics version 27.0, GraphPad Prism version 6.0, and the R programming language. Categorical variables were summarized as absolute frequencies and percentages and, where appropriate, compared using the chi-square test or Fisher’s exact test. Quantitative variables were summarized using measures of central tendency and dispersion appropriate for their distributions. All statistical tests were two-sided, and *p*-values < 0.05, or adjusted *p*-values when for multiple comparisons correction was applied, were considered statistically significant, as previously described [29,55,56].

### 4.3. New Alleles Described in URDs Registry of Murcia Region

We also analyzed the new HLA alleles described in our region using SBT (2018–2021) and NGS techniques (2021–2025) in two ways: with respect to the HLA loci involved and with respect to the exons, introns, or 3’UTR where they were detected, as previously published [14,15,17,57,58,59,60,61]. It is important to note that our population has been studied across different genes and processes [20,29,62,63,64].

Finally, regarding the new alleles identified in the Murcia region (*n* = 39) compared to the total number of alleles discovered in Spain (*n* = 1920), they represent 2% of that total. However, it should be noted that for a long time, the definition of new alleles was carried out exclusively in Madrid and Barcelona (data obtained from the IMGT/HLA website: https://www.ebi.ac.uk/ipd/imgt/hla/about/statistics/world/ (accessed on 14 September 2025) [65]. Regarding the situation in the rest of Europe, there is a gradient, with high numbers of new alleles reported by Germany (*n* = 11.559), followed by the UK (*n* = 6.345). Other countries fall into a different tier, such as France (*n* = 2.207), Spain (*n* = 1.920), Russia (*n* = 1.071), and The Netherlands (*n* = 928). There is then a significant drop to Italy (*n* = 416), followed by smaller numbers in Greece (*n* = 145), Belgium (*n* = 135), Croatia (*n* = 129), Sweden (*n* = 128), Switzerland (*n* = 70), and Austria (*n* = 68).

Finally, there are entire countries with fewer reports of new alleles than the Murcia region itself (*n* = 39), such as Portugal (*n* = 30), Denmark (*n* = 29), Ireland (*n* = 26), the Czech Republic (*n* = 16), Lithuania (*n* = 13), Norway (*n* = 12), Finland (*n* = 5), and Bulgaria (*n* = 4). Serbia, Romania, and Hungary are among those with only a single new allele reported [65,66,67,68,69,70,71]. This highlights the excellent work carried out in this region to define and advance the study of HLA polymorphism.

## Figures and Tables

**Table 1:** Demographic data and characteristics of URD recruited between 1992 and 2025, by sex and age group.

	Absolute Number	Percent (%)
Inclusion by male sex	12.178	36.6
Inclusion by female sex	21.093	63.4
Inclusion by age group 18–30 ^a^	11.782	35.51
Inclusion by age group 31–40 ^a^	10.668	32.06
Inclusion by age group 41–50 ^a^	7.061	21.22
Inclusion by age group 51–55 ^a^	3.760	11.30

URD, unrelated donors: ^a^ This refers to age at the time of inclusion. Comparisons were performed using Fisher’s exact test or the chi-square test for qualitative variables, and the nonparametric Mann–Whitney U test for quantitative variables. *p*-values < 0.05 were considered significant.

**Table 2:** Observed and expected heterozygosity and Hardy–Weinberg equilibrium analysis of HLA loci in the Murcia unrelated donor population.

Locus	*n*	Observed Heterozygosity	Expected Heterozygosity	*p*-Value	SD	Steps Done
HLA-A	650	0.82812	0.87808	0.10679	0.00010	10,100
HLA-B	650	0.98312	0.96432	0.43422	0.00009	10,100
HLA-C	650	0.87500	0.92975	0.07920	0.00008	10,100
HLA-DRB1	650	0.96875	0.94870	0.12968	0.00008	10,100
HLA-DQB1	650	0.92188	0.90859	0.40191	0.00035	10,100
HLA-DPB1	650	0.81250	0.85322	0.03526	0.00010	10,100

HWE, Hardy–Weinberg equilibrium; NGS, next-generation sequencing; SD, standard deviation.

**Table 3:** Expected heterozygosity of HLA loci in the Murcia URD population.

Locus HLA	He
HLA-A	0.87808
HLA-B	0.96432
HLA-C	0.92975
HLA-DRB1	0.94870
HLA-DQB1	0.90859
HLA-DPB1	0.85322
Mean	0.91378

URD, unrelated donors; He, expected heterozygosity.

**Table 4:** Allele frequencies of HLA class I loci in the Murcia unrelated donor population (*n* = 650).

HLA-A	Frequency (%)	HLA-B	Frequency (%)	HLA-C	Frequency (%)
A*01:01	10.94	B*07:02	5.4	C*01:01	2.3
A*01:02	1.5	B*08:01	7.8	C*01:02	4.9
A*02:01	30.46	B*13:02	1.5	C*02:02	0.7
A*02:02	1.5	B*14:01	1.9	C*02:10	0.7
A*02:05	2.3	B*14:02	3.1	C*03:03	2.3
A*02:19	0.7	B*15:01	3.1	C*03:04	2.3
A*03:01	7.8	B*15:03	0.7	C*04:01	14.1
A*03:02	0.7	B*15:04	0.7	C*05:01	10.9
A*11:01	3.9	B*15:24	0.7	C*06:02	8.2
A*23:01	3.1	B*18:01	4.7	C*07:01	14.1
A*24:02	7.8	B*27:05	2.3	C*07:02	7.0
A*24:03	1.5	B*27:12	0.7	C*07:04	0.7
A*25:01	7.8	B*35:01	3.1	C*07:06	0.7
A*26:01	1.5	B*35:02	1.5	C*07:18	0.7
A*29:01	1.5	B*35:03	2.3	C*08:02	3.5
A*29:02	7.8	B*35:08	1.5	C*12:02	2.3
A*30:01	0.7	B*37:01	0.7	C*12:03	4.7
A*30:02	2.3	B*38:01	2.3	C*14:02	1.5
A*30:04	0.7	B*39:01	1.5	C*14:03	0.7
A*31:01	1.5	B*39:05	0.7	C*15:02	2.3
A*32:01	1.5	B*39:06	0.7	C*15:05	0.7
A*33:01	2.3	B*39:10	0.7	C*16:01	6.2
A*33:03	0.7	B*39:12	0.7	C*16:02	0.7
A*36:01	0.7	B*40:01	3.1	C*17:01	0.7
A*43:01	0.7	B*40:02	1.5	C*17:02	0.7
A*66:01	0.7	B*41:01	1.5	C*18:01	0.7
A*66:02	0.7	B*42:01	0.7		
A*68:01	1.5	B*44:02	10.1		
A*68:02	4.6	B*44:03	8.6		
A*68:17	0.7	B*44:05	0.7		
A*69:01	0.7	B*45:01	3.1		
A*74:01	0.7	B*47:01	0.7		
A*80:01	0.7	B*48:01	0.7		
		B*49:01	6.8		
		B*50:01	1.5		
		B*51:01	5.3		
		B*52:01	1.5		
		B*53:01	2.3		
		B*54:01	0.7		
		B*55:01	0.7		
		B*56:01	0.7		
		B*57:01	2.3		
		B*58:01	1.5		
		B*59:01	0.7		
		B*73:01	0.7		

Only alleles detected in at least two unrelated donors or in one URD with clear four-digits difference were reported. The asterisk (*) is part of the HLA allele nomenclature and separates the locus designation from the allele designation.

**Table 5:** Allele frequencies of HLA class II loci in the Murcia unrelated donor population (*n* = 650).

HLA-DRB1	Frequency (%)	HLA-DQB1	Frequency (%)	HLA-DPB1	Frequency (%)
DRB1*01:01	5.9	DQB1*02:01	12.5	DPB1*01:01P	3.9
DRB1*01:02	3.1	DQB1*02:02	12.9	DPB1*02:01P	10.2
DRB1*01:03	0.7	DQB1*03:01	17.9	DPB1*02:02P	3.1
DRB1*03:01	11.7	DQB1*03:02	10.1	DPB1*03:01P	9.8
DRB1*03:02	0.7	DQB1*03:03	0.7	DPB1*04:01P	35.3
DRB1*04:01	1.5	DQB1*03:04	0.7	DPB1*04:02P	10.3
DRB1*04:02	0.7	DQB1*03:05	0.7	DPB1*05:01P	1.5
DRB1*04:03	3.9	DQB1*03:09	0.7	DPB1*06:01P	1.5
DRB1*04:04	3.1	DQB1*03:19	2.3	DPB1*09:01P	0.7
DRB1*04:05	2.3	DQB1*04:01	0.7	DPB1*10:01P	3.1
DRB1*04:07	1.5	DQB1*04:02	6.2	DPB1*11:01P	4.7
DRB1*07:01	13.5	DQB1*05:01	10.2	DPB1*13:01P	1.5
DRB1*08:01	3.1	DQB1*05:02	1.5	DPB1*14:01P	0.7
DRB1*08:02	1.5	DQB1*05:03	1.5	DPB1*15:01P	1.6
DRB1*09:01	0.7	DQB1*05:04	0.7	DPB1*16:01P	0.7
DRB1*10:01	0.7	DQB1*06:01	3.9	DPB1*17:01P	5.4
DRB1*11:01	5.5	DQB1*06:02	5.5	DPB1*18:01P	0.7
DRB1*11:02	2.3	DQB1*06:03	9.3	DPB1*19:01P	0.7
DRB1*11:03	0.7	DQB1*06:04	3.9	DPB1*20:01P	0.7
DRB1*11:04	5.4	DQB1*06:09	0.7	DPB1*23:01P	0.7
DRB1*12:01	0.7			DPB1*26:01P	0.7
DRB1*13:01	8.5			DPB1*30:01P	0.7
DRB1*13:02	5.4			DPB1*34:01P	0.7
DRB1*13:03	2.3			DPB1*35:01P	0.7
DRB1*13:05	0.7			DPB1*45:01P	0.7
DRB1*14:01	0.7			DPB1*63:01P	0.7
DRB1*14:54	1.5			DPB1*104:01P	1.5
DRB1*15:01	6.3			DPB1*124:01P	0.7
DRB1*15:02	1.5			DPB1*126:01P	0.7
DRB1*16:01	1.5			DPB1*133:01P	0.7
DRB1*16:02	0.7			DPB1*158:01P	0.7

Only alleles detected in at least two URDs or in one URD with clear four-digits difference were reported. The asterisk (*) is part of the HLA allele nomenclature and separates the locus designation from the allele designation.

**Table 6:** Most frequent HLA haplotypes in the URD population.

HLA-A-C-B-DRB1-DQB1	Total Frequency ^a^ (%)	Partial Frequency ^b^ (%)
A*29:02-C*16:01-B*44:03-DRB1*07:01-DQB1*02:02	3.3	25.4
A*01:01-C*07:01-B*08:01-DRB1*03:01-DQB1*02:01	2.7	20.8
A*30:02-C*05:01-B*18:01-DRB1*03:01-DQB1*02:01	1.8	13.8
A*33:01-C*08:02-B*14:02-DRB1*01:02-DQB1*05:01	1.5	11.5
A*03:01-C*07:02-B*07:02-DRB1*15:01-DQB1*06:02	0.9	6.9
A*02:01-C*07:02-B*07:02-DRB1*15:01-DQB1*06:02	0.7	5.4
A*02:01-C*16:01-B*44:03-DRB1*07:01-DQB1*02:02	0.6	4.6
A*26:01-C*12:03-B*38:01-DRB1*13:01-DQB1*06:03	0.6	4.6
A*02:01-C*03:03-B*15:01-DRB1*04:01-DQB1*03:02	0.5	3.8
A*02:01-C*05:01-B*18:01-DRB1*03:01-DQB1*02:01	0.4	3.1

^a^ These frequencies refer to the total number of haplotypes detected in our population; ^b^ these frequencies refers to the ten frequent haplotypes of this table. The asterisk (*) is part of the HLA allele nomenclature and separates the locus designation from the allele designation.

## Data Availability

The original contributions presented in this study are included in the article. Further inquiries can be directed to the corresponding author.
